# The Gut Microbiota–Mast Cell Axis in Intestinal Homeostasis and Food Allergy Pathogenesis

**DOI:** 10.3390/biom16020254

**Published:** 2026-02-05

**Authors:** Alessia Carnevale, Caterina Marangio, Erisa Putro, Rosa Molfetta, Rossella Paolini

**Affiliations:** 1 Laboratory Affiliated to Istituto Pasteur Italia—Fondazione Cenci Bolognetti, Department of Molecular Medicine, Sapienza University of Rome, 00161 Rome, Italy; a.carnevale@uniroma1.it (A.C.); caterina.marangio@uniroma1.it (C.M.); rosa.molfetta@uniroma1.it (R.M.); 2 Department of Clinical and Molecular Medicine, Sapienza University of Rome, 00189 Rome, Italy; erisa.putro@uniroma1.it

**Keywords:** food allergy, mast cells, microbiota, dysbiosis

## Abstract

Food allergy is an increasing global health burden, particularly in industrialized countries, with rising prevalence in both pediatric and adult populations. It is characterized by exaggerated immune responses to innocuous dietary antigens, leading to clinical manifestations ranging from mild gastrointestinal symptoms to life-threatening anaphylaxis. Mast cells are central effectors in the pathophysiology of food allergy, initiating and amplifying allergic inflammation through the release of a broad array of mediators upon activation. Recent studies have revealed that the intestinal microbiota plays a critical role in shaping immune responses, including the regulation of mast cell development, maturation, and activation. Moreover, dysbiosis has been associated with increased susceptibility to allergic sensitization and heightened mast cell reactivity. This review explores the molecular mechanisms underlying the microbiota–mast cell axis in the context of intestinal homeostasis and food allergy with a particular emphasis on the regulation of mast cell effector functions by TLR signaling and microbial metabolites. We also discuss the therapeutic potential of targeting the microbiota–mast cell axis as novel strategies to restore immune tolerance. Understanding this complex crosstalk opens new avenues for translational approaches in the prevention and treatment of food allergy.

## 1. Introduction

The prevalence of food allergy (FA) has increased dramatically over the past decades, reflecting not only genetic predisposition but also profound changes in environmental exposures, lifestyle, and dietary habits. Early-life microbial colonization plays a pivotal role in the education of the immune system, influencing the development of oral tolerance and shaping the susceptibility to allergic diseases. Disruptions in the gut microbiota—whether due to cesarean delivery, antibiotic exposure, or Western-style diets—can compromise epithelial barrier integrity, alter antigen sampling, and promote inappropriate mast cell activation, establishing a feed-forward loop that sustains allergic inflammation. Recent advances have highlighted the dynamic interactions between microbial communities, epithelial cells, and mast cells, revealing how microbial metabolites and innate immune signaling modulate mast cell function and mucosal homeostasis. In this review, we synthesize current knowledge on the microbiota–epithelium–mast cell axis, highlighting mast cells as central effector cells in the pathogenesis of FA and underscoring how targeting mast cell–microbiota interactions may offer promising translational strategies to restore mechanisms of mucosal tolerance.

## 2. Unraveling the Mechanistic Basis of Food Allergy

Allergies are pathological immune reactions elicited by otherwise harmless proteins or protein-associated substances. Among these conditions, FAs represent a major subgroup of type I (immediate) hypersensitivity disorders, which also include asthma, allergic rhinitis, atopic dermatitis, and drug hypersensitivity [1]. FAs occur when ingestion of a normally innocuous dietary antigen elicits an aberrant immune response, resulting in clinical manifestations that range from mild, localized symptoms to severe, systemic, and potentially life-threatening reactions. The most common food allergens worldwide include cow’s milk, hen’s egg, peanut, tree nuts, soy, wheat, fish, and shellfish, which together account for most clinically relevant reactions [2,3]. For many years, FA was considered predominantly a paediatric condition, as it often manifests early in life and frequently resolves with age, particularly in the case of cow’s milk and hen’s egg allergy. Peanut allergy represents a notable exception, as it commonly persists into adulthood [3]. In individuals with an atopic predisposition, FA develops when oral tolerance fails to be established or is disrupted due to alterations in intestinal immune homeostasis. This loss of physiological tolerance is often initiated by epithelial damage and the release of soluble mediators that generate a pro-inflammatory microenvironment, favouring a Th2-skewed immune response. Dietary allergens can penetrate a compromised epithelial barrier, a process facilitated by genetic defects in structural proteins such as filaggrin [4], as well as by environmental stressors. Specialized microfold (M) cells transport luminal antigens to underlying immune cells, including dendritic cells (DCs). Among these, CD103^+^ DCs—normally involved in the induction of regulatory T (Treg) cells and the maintenance of tolerance—acquire a Th2-promoting phenotype in FA. In parallel, CX3CR1^+^ DCs and resident macrophages capture luminal antigens and interact with Th2 lymphocytes and type 2 innate lymphoid cells (ILC2s), thereby amplifying early Th2-driven inflammation [5]. Under the influence of epithelial-derived alarmins such as thymic stromal lymphopoietin (TSLP), interleukin (IL)-25, and IL-33, allergen-loaded DCs upregulate co-stimulatory molecules while suppressing tolerogenic signals, including IL-10, transforming growth factor-β (TGF-β), and retinoic acid (RA) [6]. These activated DCs migrate to the mesenteric lymph nodes, where allergen-derived peptides are presented on major histocompatibility complex class II molecules to naïve CD4^+^ T cells. In the presence of a Th2-polarizing cytokine milieu, these T cells differentiate into Th2 effector cells [5]. Th2 cells secrete IL-4, IL-5, and IL-13, promoting IgE class-switch recombination in B cells, eosinophil recruitment, mucus production, and tissue remodeling [7]. Allergen-specific IgE binds with high affinity to FcεRI receptors expressed on mast cells (MCs) and basophils, sensitizing these cells without eliciting clinical symptoms at this stage [8]. Upon allergen re-exposure, FcεRI crosslinking triggers Lyn–Syk-dependent signaling, leading to rapid degranulation and release of histamine and proteases, as well as to the synthesis of lipid mediators (leukotrienes and prostaglandin D_2_), which together drive the acute manifestations of IgE-mediated allergy [9]. Cutaneous manifestations are among the most frequent clinical features of FA, reflecting the high density of MCs in the skin, particularly within the dermis and perivascular regions. Upon allergen exposure, MC degranulation leads to vasodilation, increased vascular permeability, and sensory nerve activation, which clinically manifest as urticaria, angioedema, erythema, and intense pruritus, typically developing within minutes [3]. Beyond the skin, mediator release also affects the gastrointestinal tract, where smooth muscle contraction and mucosal edema give rise to symptoms such as vomiting, abdominal pain, and diarrhea [3]. Respiratory involvement may further complicate the clinical picture, impacting both the upper and lower airways and resulting in sneezing, rhinorrhea, bronchospasm, mucus hypersecretion, and airway edema [3]. Notably, in the context of seafood allergy, respiratory symptoms may occur not only after ingestion but also following inhalation of aerosolized allergens produced during cooking or processing [10]. In severe cases, the systemic dissemination of mediators can impair cardiovascular function, leading to hypotension, tachycardia, and ultimately anaphylaxis [3]. MC-derived cytokines also initiate a late-phase reaction occurring 2–24 h after allergen exposure, characterized by the recruitment of eosinophils, neutrophils, and additional Th2 lymphocytes. This sustained inflammatory response contributes to tissue damage, vascular leakage, and gastrointestinal dysfunction. Recurrent exposure and repeated late-phase responses may promote chronic allergic inflammation, in which both Th2- and Th1-associated cytokines coexist, driving tissue remodeling and persistent barrier disruption. In the most severe cases, massive mediator release can result in airway obstruction, cardiovascular collapse, and fatal shock [11].

MCs are increasingly recognized as multifunctional immune sentinels whose activation extends beyond classical IgE-dependent pathways. Their broad repertoire of surface and intracellular receptors enables them to integrate immunological and non-immunological signals, including cytokines, alarmins, microbial products, and neuroimmune stimuli. This functional plasticity underlies their involvement across a spectrum of allergic and inflammatory disorders, ranging from purely IgE-mediated conditions to mixed and IgE-independent phenotypes. Mixed IgE/non-IgE-mediated diseases combine immediate IgE-driven responses with delayed, cell-mediated inflammation. Eosinophilic esophagitis (EoE) exemplifies this interplay, in which IL-13-mediated epithelial dysfunction and eotaxin-3-dependent eosinophil recruitment converge with tissue remodeling orchestrated by MCs and ILC2s [12,13]. Beyond IgE-mediated and mixed mechanisms, IgE-independent FAs constitute a distinct group of immune-mediated disorders driven by alternative cellular pathways. In these conditions, epithelial barrier disruption and inflammatory cues promote Th1, Th17, and cytotoxic T-cell responses while suppressing tolerogenic mechanisms, resulting in characteristic inflammatory patterns and tissue injury [14]. A major unresolved question is how distinct microbial communities mechanistically shape MC activation in these contexts. Although dysbiosis in mixed IgE/non-IgE disorders appears qualitatively distinct from that observed in IgE-mediated FA [15,16], direct causal links between defined microbial signatures, MC subset activation, and mediator release remain to be established. Collectively, these examples highlight the diverse immunological pathways through which food allergens disrupt physiological homeostasis, underscoring the central role of MCs alongside innate and adaptive immune components in shaping clinical outcomes.

## 3. Microbiota–Mast Cell Interactions in Gut Homeostasis

### 3.1. The Gut Microbiota–Immune Axis in Intestinal Homeostasis

The gut microbiota comprises a highly diverse and dynamic microbial community that colonizes the human gastrointestinal tract and plays a central role in both intestinal and systemic homeostasis. Consisting of approximately 100 trillion microorganisms, the gut microbiota represents the most influential microbial ecosystem in the human body with respect to immune regulation and host physiology [17,18]. A healthy microbiota is characterized by high taxonomic diversity and a relatively stable core dominated by the phyla *Firmicutes* and *Bacteroidetes*, accompanied by a variable component that enables adaptation to dietary and environmental cues [19,20]. Living in close symbiosis with the host, these microbial communities are essential for maintaining intestinal homeostasis and immune balance at the mucosal interface [21]. The spatial organization of the gut microbiota along the intestinal tract critically shapes its functional specialization. The small intestine, characterized by rapid transit, high bile acid concentrations, and relatively elevated oxygen levels, **harbours** a low-diversity microbial community enriched in aerobes and facultative anaerobes. In contrast, the colon provides an anaerobic, nutrient-rich environment that sustains high microbial density and diversity, predominantly composed of obligate anaerobes [17,18,19]. This spatial compartmentalization underpins the distinct metabolic and immunological functions exerted by the microbiota along the gut. Through continuous interactions with the host, the gut microbiota forms an integrated functional unit with epithelial and immune cells that preserves mucosal integrity and prevents inappropriate immune activation [22]. The intestinal epithelium provides a physical and biochemical barrier composed of specialized cell types, including goblet cells that produce MUC2-rich mucus, thereby creating a protective niche for commensal microorganisms, and Paneth cells that secrete antimicrobial peptides limiting microbial translocation [23,24,25]. In parallel, plasma cell-derived secretory IgA coats luminal bacteria, shaping microbial composition and restricting antigen penetration [26]. Beyond these well-established barrier mechanisms, emerging evidence indicates that pigmented cells, including melanocytes, may be present in the gut mucosa and Peyer’s patches, where they could contribute to local antimicrobial defense and potentially influence microbial colonization and mucosal immune responses [27].

Signals derived from commensal microorganisms are continuously sensed by immune cells within the gut-associated lymphoid tissue through pattern-recognition receptors, enabling discrimination between harmless symbionts and potential pathogens [28]. This microbial sensing fosters immune tolerance by promoting regulatory T-cell differentiation and the production of anti-inflammatory cytokines such as IL-10 and TGF-β, whereas disruption of this **dialogue** leads to dysbiosis and chronic intestinal inflammation [29]. The essential contribution of the microbiota to immune maturation is underscored by germ-free models, which exhibit impaired development of Peyer’s patches, reduced numbers of IgA-producing plasma cells and CD4^+^ T cells, and disorganized secondary lymphoid structures; these defects are largely reversed upon microbial recolonization [30]. Beyond immune education, the gut microbiota confers colonization resistance against pathogens through direct competition for nutrients and adhesion sites and through the production of antimicrobial factors. Indirectly, it reinforces epithelial defenses by enhancing mucus secretion, antimicrobial peptide release, and secretory IgA production [31,32]. Collectively, these protective mechanisms highlight the role of the microbiota as an active and dynamic component of the intestinal barrier. In addition to structural and immunological interactions, microbial metabolism represents a key axis of host–microbiota crosstalk. By fermenting indigestible dietary substrates, gut bacteria generate metabolites with profound effects on epithelial function and immune regulation [33]. Short-chain fatty acids (SCFAs), including acetate, propionate, and butyrate, serve as central mediators of this communication. Acetate and propionate regulate hepatic lipid and glucose metabolism, whereas butyrate acts as the primary energy source for colonocytes and promotes epithelial barrier integrity [34,35]. Moreover, SCFAs modulate mucosal immunity by enhancing mucin and secretory IgA production and by reinforcing epithelial tight junctions through G protein–coupled receptor signaling [36,37,38,39]. Additional microbial metabolites further shape intestinal homeostasis. Secondary bile acids, generated through microbial conversion of primary bile acids, signal to restrain inflammatory responses [40,41,42]. Tryptophan-derived indole metabolites activate the aryl hydrocarbon receptor in epithelial and immune cells, promoting IL-22 production, antimicrobial peptide expression, mucin secretion, and epithelial barrier reinforcement while concurrently inhibiting NF-κB signaling [43,44].

Beyond their effects on epithelial and immune compartments, microbial metabolites also influence the enteric nervous system (ENS), thereby modulating gut motility, secretion, and neuroimmune interactions. ENS neurons and glial cells sense microbial-derived signals and luminal antigens and respond by releasing neuropeptides such as vasoactive intestinal peptide and calcitonin gene-related peptide. These mediators regulate MCs, DCs, and lymphocytes, establishing a functional link within the microbiome–gut–brain axis [45]. The gut microbiota further communicates with the ENS and the central nervous system through nitric oxide (NO) signaling. Microbial metabolites and dietary antigens modulate NO production in non-adrenergic non-cholinergic neurons via neuronal nitric oxide synthase, whereas Th2-driven inflammation upregulates inducible nitric oxide synthase during FA, thereby influencing barrier integrity, immune cell recruitment, and microbial interactions [46]. Collectively, NO emerges as a key mediator linking microbial metabolism, mucosal immunity, and neuronal regulation, adding an additional layer of control over intestinal homeostasis.

Together, these interconnected pathways illustrate how the gut microbiota orchestrates epithelial, immune, and neuronal functions to maintain intestinal homeostasis.

### 3.2. Microbiota–Mast Cell Crosstalk in the Regulation of Intestinal Immunity

**Figure 1:**
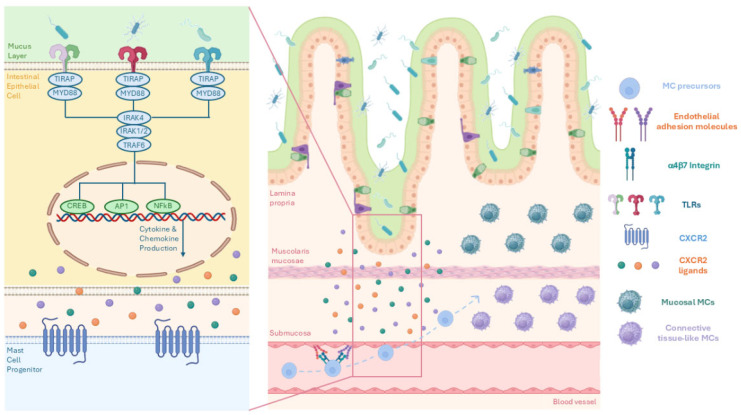
Microbiota-dependent recruitment and compartmentalization of mast cells in the intestine. Gut microbiota is essential to promote the maturation of the intestinal mast cell (MC) progenitors and to ensure normal physiological function [50]. The gut microbiota activates Toll-like receptor (TLR) signaling in intestinal epithelial cells, leading to MyD88-dependent activation of downstream kinases (IRAKs, TRAF6) and transcription factors, including NFκB, AP-1, and CREB, resulting in the production of cytokines and chemokines, notably CXCR2 ligands [49]. MC progenitor homing is mediated by the interaction of α4β7 integrin expressed on progenitors with endothelial adhesion molecules, including MAdCAM-1, ICAM-1, and VCAM-1, and is further modulated by CXCR2-dependent chemotactic signaling [49]. Following transmigration from the bloodstream into the intestinal tissue, MC progenitors differentiate into distinct MC subsets occupying specific anatomical niches. Mucosal mast cells (MMCs) are predominantly localized within the lamina propria and are characterized by the expression of mMCP-1 and mMCP-2, whereas connective tissue-like mast cells (CTMCs) reside mainly in the submucosa and express mMCP-4, -5, -6, and -7 [51]. Collectively, the figure illustrates how microbiota-driven epithelial signaling regulates intestinal MC recruitment, localization, and functional heterogeneity. Figure created in BioRender. Alessia Carnevale. (2025) https://BioRender.com.

MCs are long-lived, granule-containing immune cells widely distributed throughout vascularized tissues, with preferential localization at mucosal interfaces—such as the skin, respiratory tract, genitourinary tract, and intestinal mucosa—where they function as sentinels at the interface between the host and the external environment [47,48]. Notably, the gastrointestinal tract harbors the largest MC population in the human body. In the intestine, the recruitment of circulating MC progenitors is primarily mediated by interactions between α4β7 integrin and endothelial adhesion molecules, including ICAM-1, VCAM-1, and MAdCAM-1, and is further modulated by CXCR2-dependent signaling [49]. The gut microbiota also contributes to MC recruitment by inducing the expression of CXCR2 ligands on intestinal epithelial cells in a MyD88-dependent manner (Figure 1). Consistently, germ-free mice exhibit a markedly reduced frequency of intestinal MCs compared with wild-type controls [50]. Within the gastrointestinal wall, mature MCs are heterogeneous, comprising two principal subsets that occupy distinct intestinal regions and differ in protease content [51]. In mice, mucosal MCs (MMCs) are abundant in the lamina propria and express mMCP-1 and mMCP-2, whereas connective tissue-like MCs (CTMCs), located in the submucosa, often in proximity to sensory fibers of the enteric nervous system, express mMCP-4, -5, -6, and -7 [51] (Figure 1). In humans, MCs are similarly classified into tryptase-positive (MCT) and tryptase–chymase double-positive (MCTC) subtypes, corresponding to murine mucosal and connective tissue-like MCs, respectively [52]. Beyond this classical dichotomy, MCs display remarkable phenotypic and functional plasticity, continuously adjusting their molecular profiles and effector functions in response to the surrounding microenvironment [53,54]. The anatomical complexity of the intestine and its dynamically changing luminal environment further shape MC heterogeneity, influencing both their molecular signature and biological activity. Through the mediators they release, intestinal MCs perform diverse functions that contribute to the maintenance of epithelial barrier integrity and overall mucosal homeostasis [55].

Notably, the ability of MCs to rapidly sense and respond to specific triggers, including neuropeptides, underlies their activated status in various human gastrointestinal disorders, such as celiac disease, irritable bowel syndrome, and inflammatory bowel disease [56,57]. In the context of food allergy, expansion of the MC compartment—driven predominantly by an increase in intestinal MMCs—has been documented in both humans and mice sensitized to dietary antigens, and this expansion correlates with symptom severity [58,59]. Mechanistic studies using two widely employed models of IgE-mediated food allergy demonstrated that systemic anaphylaxis is specifically linked to activation of connective tissue-like MCs, whereas gastrointestinal manifestations are characterized by an expansion of mMCP-1-expressing MMCs accompanied by activation of both mucosal and connective tissue-like MC subsets [60]. More recently, depletion of the Mrgprb2^+^ CTMC subset was shown to protect mice from anaphylactic shock, while Mrgprb2^−^ gut MMCs do not contribute to systemic responses [61].

**Figure 2:**
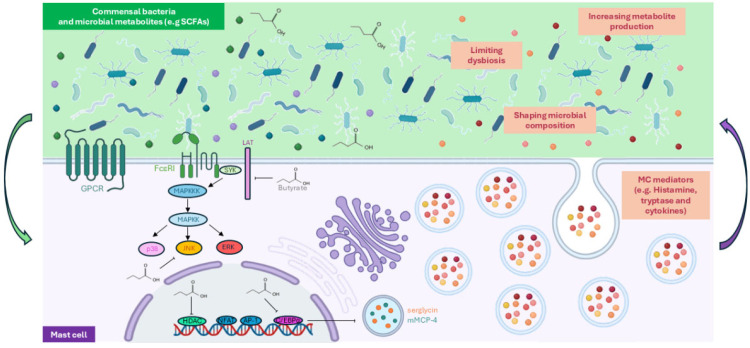
Bidirectional microbiota–mast cell crosstalk in the gut homeostasis. Commensal bacteria produce microbial metabolites that continuously modulate mast cell (MC) activation in the intestine. Short-chain fatty acids (SCFAs), particularly butyrate, signal through G protein-coupled receptors (GPCR) on MCs [64]. Butyrate acts as a key epigenetic regulator by inhibiting histone deacetylases, repressing MC activation programs and dampening FcεRI-dependent signaling, selectively reducing JNK activation, MC degranulation, proliferation, and mediator release [65,66,67,68]. Conversely, MC derived mediators, such as histamines, tryptases, and cytokines, are pivotal in maintaining gut homeostasis by shaping microbial composition and limiting dysbiosis [70,71,72]. Through this bidirectional communication, MCs integrate microbial and host-derived signals to maintain mucosal homeostasis, with MC activation state and effector repertoire determining their impact on intestinal immunity. Figure created in BioRender. Alessia Carnevale. (2025) https://BioRender.com.

MCs and the gut microbiota exist in a state of constant bidirectional communication, forming a dynamic axis that supports mucosal homeostasis by balancing immune tolerance and defense (Figure 2). Commensal bacteria—including non-pathogenic *Escherichia* *coli*, *Enterococcus faecalis*, and various *Lactobacillus* species—suppress MC degranulation in response to IgE or secretagogue stimuli without affecting FcεRI expression, thereby limiting unnecessary inflammation [62,63]. Microbial metabolites, including acetate, propionate, butyrate, and valerate, further modulate MC function through G protein-coupled receptors [64]. Butyrate alters histone acetylation at promoters and super-enhancers of critical MC genes, repressing activation programs, suppressing proliferation, histamine release, and cytokine production via histone deacetylase inhibition, reducing JNK phosphorylation while sparing ERK1/2 and p38, dampening FcεRI-dependent signaling by inhibiting BTK, SYK, and LAT phosphorylation, and restricting MC expansion by downregulating c-Kit signaling [65,66,67]. Butyrate also upregulates C/EBPα, which represses serglycin and mMCP-4, thereby reducing granule mediator storage [68]. Overall, butyrate functions as a key epigenetic regulator mainly via histone deacetylase inhibition, although potential differences in its effects among intestinal MC subsets remain to be elucidated. Other microbial metabolites, such as hydrogen sulfide (H_2_S) produced by colonic bacteria, contribute to mucosal protection by suppressing MC degranulation [69].

Conversely, MCs actively influence the gut microbial ecosystem (Figure 2). In murine models of *Candida albicans* colonization, MC deficiency leads to inflammatory dysbiosis, characterized by expansion of *Clostridiaceae* followed by *Enterobacteriaceae*, correlating with heightened intestinal inflammation and impaired microbiota-mediated antifungal defense [70]. Similarly, FcεRIα-deficient mice exhibit attenuated DSS-induced colitis, demonstrating that specific MC signaling axes contribute to disease severity. This protection is microbiota-dependent, as the absence of FcεRIα selectively enriches *Lactobacillus plantarum*, enhancing production of lactic acid, a microbial metabolite with anti-inflammatory properties [71]. Furthermore, deficiency of mMCP-4 exacerbates DSS-induced inflammation, driving marked alterations in the colonic microbiota and reducing the generation of anti-inflammatory metabolites, with consequent impairment of epithelial barrier function [72]. Collectively, these studies establish MCs as central regulators of gut microbial homeostasis and intestinal immunity.

## 4. The Microbiota in the Pathogenesis of Food Allergy

The development of FA reflects a complex interplay of genetic, environmental, and immunological factors that remain incompletely understood. The marked increase in FA prevalence over recent decades—particularly in industrialized regions—represents a major public health concern, as strict allergen avoidance remains the only effective therapeutic strategy. This rise parallels the increasing burden of other Western-associated inflammatory and metabolic disorders, including obesity and inflammatory bowel disease, suggesting shared environmental drivers [3]. The rapidity of this epidemiological shift cannot be explained by genetic change alone, highlighting a central role for environmental influences. Early models, such as the hygiene hypothesis, proposed that reduced exposure to childhood infections predisposes to atopy [73]. More recent frameworks extend this concept by emphasizing the loss of environmental and microbial diversity associated with industrialized lifestyles. In particular, the “old friends” and “biodiversity” hypotheses posit that reduced exposure to commensal, soil-derived, and animal-associated microorganisms impairs the development of immunoregulatory networks shaped over human evolution [74,75]. Consistent with these models, urbanization, widespread antibiotic use, and low-fiber, highly processed diets disrupt gut microbiota composition and function [76,77], whereas populations living in rural or farming environments, as well as communities chronically exposed to helminths, exhibit lower rates of allergic sensitization and FA [78]. Together, these observations implicate microbiota-mediated impairment of mucosal tolerance as a key driver of FA susceptibility, with disease manifestation and severity shaped by allergen properties, host genetics, epithelial barrier integrity, and gut microbial composition.

Importantly, the establishment of these host–microbe interactions occurs predominantly early in life, during a critical window of immune development. Mode of delivery, antibiotic exposure, infant feeding practices, and early dietary patterns collectively shape the taxonomic and functional maturation of the infant gut microbiota, biasing immune development toward either tolerance or allergic sensitization [79,80]. Prospective human cohort studies indicate that microbial dysbiosis precedes FA onset, supporting a contributory role in disease development [81]. Infants who later develop FA exhibit delayed colonization by *Bacteroidetes* and *Clostridia*, reduced abundance of short-chain fatty acid (SCFA)-producing taxa—including *Bifidobacterium*, *Lactobacillus*, *Faecalibacterium prausnitzii*, and *Clostridia clusters IV* and *XIVa*—and expansion of pathobionts such as *Enterobacteriaceae*, *Escherichia–Shigella*, and *Ruminococcus gnavus* [82,83]. Despite substantial variability in taxon-level associations across cohorts—driven by differences in population characteristics, developmental timing, and analytical approaches—functional analysis reveals a striking convergent, marked by impaired fiber fermentation, reduced SCFA production, and compromise induction of regulatory immune pathways during early life [84]. Longitudinal analyses identify the first 3–6 months of life as a critical window during which gut colonization supports RORγt^+^ regulatory T-cell differentiation, IgA–microbiota interactions, and oral tolerance [85]. Within this window, *Clostridia*-enriched microbial signatures associate with FA resolution, whereas persistent dysbiosis correlates with disease persistence [83,86]. To move beyond association and establish causality, mechanistic insights have largely been derived from gnotobiotic and murine models. Transplantation of microbiota from healthy infants—but not from infants with cow’s-milk allergy—confers protection against allergic sensitization in germ-free mice, identifying butyrate-producing *Clostridiales* as key mediators of tolerance [87,88]. Colonization with murine or human-derived *Clostridium* species suppresses allergen-specific IgE production and prevents anaphylaxis in vivo [89,90]. Protection is mediated in part through IL-22-dependent epithelial reinforcement driven by ILC3 activation, enhancing mucus secretion, antimicrobial peptide production, and barrier integrity [91], thereby limiting systemic allergen uptake and supporting the “barrier regulation hypothesis” [92]. Conversely, germ-free or antibiotic-treated mice exhibit exaggerated IL-4 responses, polyclonal IgE production, and heightened susceptibility to anaphylaxis, which can be transmitted via microbiota from sensitized *Il4raF709* mice [93,94]. While findings from murine and gnotobiotic models must be interpreted with caution given fundamental immunological and physiological differences between mice and humans, their integration with longitudinal human cohort studies provides converging evidence for a central, time-sensitive role of early-life microbiota composition and function in shaping mucosal tolerance, and thereby influencing FA susceptibility, persistence, and resolution. Collectively, these findings indicate that alterations in early-life microbiota composition and function are not merely associative but exert causal effects on mucosal tolerance. At the mechanistic level, dysbiosis perturbs immune–epithelial homeostasis through multiple, interlinked pathways. Of note, dysbiosis disrupts immune–epithelial homeostasis by reducing IL-10 in CX3CR1^+^ mononuclear phagocytes, impairing retinoic acid (RA) generation in stromal niches, skewing Treg subsets toward dysfunctional GATA-3^+^ Th2-like profiles, and promoting IgE class switching [95,96,97]. Microbial metabolites—including SCFAs—counteract these perturbations by supporting RORγt^+^ Treg induction, regulating dendritic-cell function, and enhancing epithelial RA/TGF-β production [98,99,100,101,102]. A central mechanistic link between dysbiosis and allergic sensitization is loss of epithelial barrier integrity. Reduced SCFAs destabilize tight-junctions (TJs) and increase claudin-2 affecting gut permeability [103]. Expansion of Gram-negative anaerobes elevates luminal LPS, driving epithelial TLR4/NF-κB activation and further compromising barrier architecture, culminating in a “leaky gut” phenotype [104,105]. Barrier breakdown permits entry of dietary allergens and microbial products, triggering release of TSLP, IL-33, and IL-25 from stressed epithelial cells, which activate Th2 cells and ILC2s [106]. These pathways amplify IL-4/IL-5/IL-13 responses, weaken TJs, and drive IgE class switching, culminating in allergen-specific IgE production and MC activation on re-exposure [107,108].

### The Microbiota–Mast Cell Axis as a Key Pathogenic Driver of Food Allergy

The gut microbiota is increasingly recognized as a key modulator of MC reactivity, thereby influencing the severity of FA. Numerous commensal and probiotic bacteria have been shown—primarily in murine models and in vitro systems—to modulate MC function by engaging inhibitory receptors such as Siglecs [109] and by attenuating FcεRI expression or signaling thresholds [110]. Indirect mechanisms also limit MC responsiveness: microbiota-driven increases in galectin-9 disrupt IgE–antigen complex formation and constrain MC degranulation [111,112], while multiple commensal species promote Foxp3^+^ regulatory T cells that suppress MC activation through OX40–OX40L interactions and bystander immunoregulatory effects [113,114]. Dietary fiber and its microbial fermentation products constitute a major axis through which the microbiota influences MC responsiveness in experimental models of FA. High-fiber diets reshape gut microbial communities, increase short-chain fatty acid (SCFA) production, and reduce serum IgE levels and anaphylaxis severity in murine models [115]. Tryptophan-derived microbial metabolites provide an additional layer of MC regulation via the aryl hydrocarbon receptor (AhR). AhR signaling is ligand- and context-dependent: acute exposure to high-affinity ligands such as FICZ enhances FcεRI-mediated degranulation and promotes IL-6 and IL-13 production in MCs, whereas sustained AhR activation induces hyporesponsiveness and protects against anaphylaxis in vivo [116,117]. AhR signaling also contributes to MC maturation and homeostasis [118]. Beyond SCFAs, structurally diverse fiber-derived oligosaccharides and polysaccharides—including prebiotics, human milk oligosaccharides, and sulfated polysaccharides—directly inhibit MC degranulation and cytokine production in experimental settings, attenuating MC-dependent allergic responses [119,120]. Although the extent to which these mechanisms operate in humans remains incompletely defined, these findings provide a mechanistic framework linking reduced dietary fiber intake, altered microbial metabolite production, dysregulated MC reactivity, and increased FA susceptibility.

**Figure 3:**
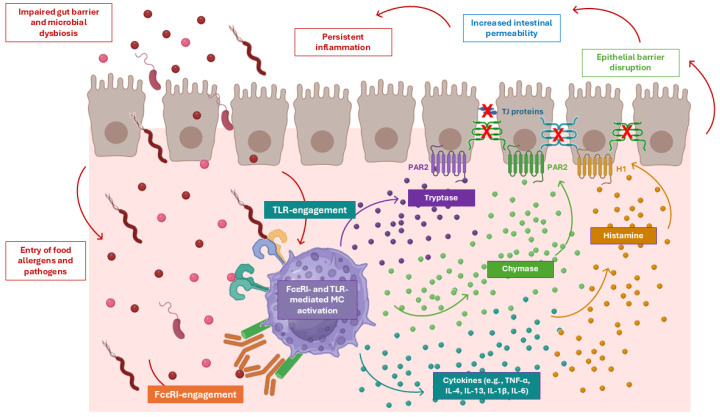
Mast cell-derived mediators disrupt epithelial barrier integrity and sustain a self-reinforcing pathogenic loop in food allergy. Mast cell (MC) activation driven by combined FcεRI- and TLR-mediated signals leads to degranulation and release of proteases, histamine, and cytokines that directly impair intestinal epithelial barrier function [124]. MC-derived proteases, including tryptase and chymase, activate protease-activated receptor 2 (PAR2) on epithelial cells, triggering signaling pathways that promote cytoskeletal contraction, degradation of tight junction (TJ) proteins, and extracellular matrix remodeling. Histamine acting through H1 receptors, together with pro-inflammatory cytokines (e.g., TNF-α, IL-4, IL-13, IL-1β, IL-6), further increases epithelial permeability [125,126,127,128,129,130,131,132,133,134]. Barrier disruption facilitates the translocation of food allergens and microbial products, perpetuating MC activation and chronic inflammation. This feed-forward loop links MC degranulation to sustained barrier dysfunction and amplification of allergic inflammation in food allergy. Figure created in BioRender. Alessia Carnevale. (2025) https://BioRender.com.

MCs are central effectors of FA through classical FcεRI crosslinking by allergen-bound IgE, but emerging evidence indicates that microbial signals from the gut microbiota can also modulate MC activation via Toll-like receptors (TLRs) [121,122]. Engagement of TLRs on MCs triggers intracellular signaling pathways that can synergize with IgE-mediated activation [123] (Figure 3). Both human and murine MCs express several TLRs—including TLR2, TLR4, TLR5, TLR7/8, and TLR9—allowing them to sense bacterial, viral, and fungal components. Among these receptors, TLR2 and TLR4 emerge as the principal mediators of synergy with FcεRI signaling, with other TLRs contributing in a context-dependent manner. TLR4 recognizes LPS from Gram-negative bacteria, whereas TLR2 senses lipoteichoic acid and peptidoglycan from Gram-positive bacteria [122]. Upon ligand binding, TLRs recruit adaptor proteins such as MyD88 (for most TLRs) or TRIF (specifically TLR3 and partially TLR4), leading to activation of downstream kinases including IRAKs, TRAF6, and TAK1. This culminates in the activation of transcription factors NF-κB, AP-1, and IRFs, orchestrating the transcription of pro-inflammatory cytokines and chemokines (e.g., TNF-α, IL-6, IL-13, CCL2) [120]. In MCs, TLR signaling also modulates Ca^2+^ mobilization and degranulation, increasing the release of histamine, proteases, and lipid mediators [124]. TLR engagement primes MCs for enhanced FcεRI-mediated degranulation, lowering the threshold for allergen-triggered responses [123]. Reduced SCFA production further contributes to MC hyperreactivity. Butyrate normally exerts epigenetic and signaling regulation, suppressing TLR-mediated cytokine production via HDAC inhibition and NF-κB repression [64,65,66,67,68]. Loss of butyrate-producing taxa removes this inhibitory checkpoint, allowing TLR signaling to proceed unchecked. Thus, the dysbiotic microbiota in FA creates a two-hit scenario for MC activation: direct allergen-driven FcεRI activation and classical degranulation, and microbe-driven TLR signaling, which lowers activation thresholds and amplifies pro-inflammatory cytokine output. This dual stimulation potentiates mucosal inflammation, promotes barrier dysfunction, and sustains the allergic phenotype, linking microbiota composition directly to MC hyperreactivity in FA. MC degranulation further exacerbates epithelial barrier disruption, perpetuating a self-reinforcing pathogenic loop (Figure 3).

MC-derived proteases—including tryptase, chymase, and carboxypeptidase A—directly compromise mucosal barrier integrity by degrading TJ proteins and extracellular matrix components. Tryptase predominantly signals through protease-activated receptor 2 (PAR2) on intestinal epithelial cells, initiating MLCK-dependent cytoskeletal contraction that redistributes ZO-1 and downregulates junctional adhesion molecule-A (JAM-A), directly impairing junctional integrity [125,126,127]. Chymase similarly activates PAR2 but signals through p38 and ERK1/2 pathways, leading to MMP-2 activation, loss of claudin-5, and proteolytic cleavage of occludin, E-cadherin, and protocadherins [128]. Thus, although tryptase and chymase share PAR2 activation, they target both overlapping and distinct TJ components, producing both convergent and complementary effects on barrier integrity. Beyond proteases, histamine acting via H1 receptors, and MC-derived cytokines—including TNF-α, IL-4, IL-13, IFN-γ, IL-1β, and IL-6—further increases intestinal epithelial permeability through discrete signaling pathways that converge on TJ remodeling and MLCK activation [129,130,131,132,133,134]. Collectively, these mediators act in parallel to selectively perturb TJ architecture, culminating in loss of barrier cohesion.

Thus, epithelial barrier dysfunction in the intestine arises from a combination of distinct and overlapping mechanisms driven by MC-derived proteases, histamine, and cytokines. This multi-layered disruption fosters persistent mucosal inflammation and compromises barrier integrity, perpetuating a self-reinforcing loop that amplifies allergic responses.

## 5. Multifaceted Approaches to Food Allergy: From Allergen Avoidance to Microbiome Modulation

FA represents the leading cause of potentially fatal anaphylaxis in industrialized countries, underscoring the urgent need for effective preventive and therapeutic strategies. While allergen avoidance remains the cornerstone of management when the causative food is identified [3], supportive interventions are necessary when the specific allergen is unknown or when ubiquitous foods such as egg or wheat are implicated. Severe reactions may require corticosteroids, but for individuals with a history of anaphylaxis, prompt intramuscular administration of epinephrine is essential at the onset of early symptoms, including pruritus, erythema, cough, or respiratory distress [3]. Epinephrine counteracts mast cell (MC)-derived bronchoconstrictive and vasodilatory mediators, increases cardiac output, and substantially reduces the risk of circulatory collapse. In milder cases, persistent cutaneous manifestations can be treated with H1-antihistamines, whereas H2-antagonists mitigate gastrointestinal symptoms mediated via gastric H2 receptors [3].

Over the past decade, significant progress has been made in allergen-specific immunotherapy, aimed at inducing immune desensitization through repeated administration of incremental allergen doses via subcutaneous, sublingual, or oral routes. These interventions reduce allergen-specific IgE levels, increase non-inflammatory IgG4 antibodies capable of competitive inhibition, and promote T-cell tolerance, ultimately rendering effector cells hyporesponsive and diminishing tissue inflammation upon allergen exposure. Immunotherapy may also shift immune polarization from a Th2- to a Th1-dominant profile and enhance regulatory T-cell differentiation [135,136]. A milestone in this field was the 2020 FDA approval of the first oral immunotherapy (OIT) for peanut allergy, with ongoing research focusing on precise mapping of IgE-binding epitopes to achieve durable or potentially permanent non-reactivity [137].

In parallel, allergen-independent biologics have emerged, including omalizumab, which binds the Fc portion of IgE, preventing its interaction with FcεRI on MCs and basophils, thereby inhibiting degranulation [138,139]. Other monoclonal antibodies target IL-5 for eosinophilic esophagitis or block IL-4 and IL-13 signaling to inhibit IgE class switching and attenuate type 2 inflammation [140,141,142,143]. DNA-based vaccines are also under investigation; in murine models, plasmid-mediated intestinal expression of Ara h 2, a major peanut allergen, confers partial protection against anaphylaxis, representing a potential approach for allergen-specific tolerance induction [144].

A growing body of evidence supports a central role for the gut microbiota in the development and maintenance of oral tolerance to dietary antigens. These insights have stimulated interest in microbiota-targeted strategies for primary prevention and as adjuncts to OIT or biologic therapies. Next-generation live biotherapeutic products (LBPs), including obligate anaerobes such as *Clostridia*, are increasingly explored due to their capacity to produce immunoregulatory metabolites such as butyrate; however, their extreme oxygen sensitivity presents formulation and delivery challenges [145]. While next-generation LBPs aim to directly reintroduce immunoregulatory microbial functions, an alternative and potentially more scalable approach focuses on selectively nurturing endogenous microbial communities through dietary substrates. Prebiotics are nondigestible substrates selectively utilized by specific members of the gut microbiota, including oligosaccharides and short-chain polysaccharides, thereby modulating microbial composition and metabolic output. Experimental and human observational studies indicate that prebiotics can promote taxa such as *Bifidobacterium*, *Lactobacillus*, *Bacteroides*, *Akkermansia*, and *Roseburia*, enhance SCFA production, support epithelial barrier function, and modulate immune responses [146,147,148,149]. Many of these immunoregulatory effects have been characterized in mechanistic and preclinical models. Prebiotics are frequently incorporated into infant formulas to approximate the functional properties of HMOs, which promote colonization with SCFA-producing bacteria, particularly those yielding acetate and butyrate that support epithelial and immune homeostasis [150,151]. In infants with suspected cow’s milk protein allergy, extensively hydrolyzed casein formulas enriched with HMOs or amino acid-based formulas supplemented with synbiotics have been shown to partially shift gut microbial profiles toward those observed in breastfed infants [152]. Maternal prebiotic supplementation during pregnancy has been reported to increase the abundance of *Bifidobacterium* in both mothers and neonates and elevate SCFA levels—effects not consistently achieved through dietary counseling alone [153,154]. However, despite strong mechanistic rationale, clinical trials evaluating prebiotics for FA prevention have yielded inconsistent results, likely reflecting substantial inter-individual variability in microbiota composition and host responsiveness. Systematic reviews conclude that current evidence remains insufficient to recommend routine prebiotic supplementation for FA prevention.

Whereas prebiotics act indirectly by shaping microbial ecology and metabolic output, probiotics seek to directly introduce live microorganisms with defined immunomodulatory properties. Probiotics can modulate immune tolerance-associated pathways through microbiota reshaping, reinforcement of epithelial barrier integrity, and activation of immunoregulatory circuits involving secretory IgA, antimicrobial peptides, and IL-10/TGF-β signaling. These mechanisms, largely characterized in preclinical and in vitro models, promote induction of Treg and contribute to the regulation of Th1/Th2 and Treg/Th17 balance [155,156,157,158,159]. In experimental models of FA, probiotic administration—most extensively studied for *Lactobacillus* and *Bifidobacterium* strains—consistently attenuates allergic phenotypes by reducing allergen-specific IgE levels, suppressing Th2 cytokine production, and enhancing tolerogenic DC and Treg responses [160,161,162]. Importantly, these effects are strongly strain-specific. Translational relevance has been investigated in human studies, with *Lactobacillus rhamnosus GG* (LGG) providing the most consistent evidence of clinical benefit. LGG supplementation has been shown to accelerate tolerance acquisition in cow’s milk allergy and to improve desensitization outcomes when combined with OIT [163,164]. Proposed mechanisms include modulation of gut microbial metabolism and butyrate-dependent epigenetic regulation of Th1/Th2- and FOXP3-associated loci; however, direct causal links in humans remain incompletely defined. Other probiotic strains have demonstrated benefits in selected clinical settings [165,166], but results across trials remain heterogeneous, underscoring the need for rigorously designed, strain-specific, and mechanistically informed clinical studies.

Given the strain-specific effects and context-dependent efficacy of probiotics, combined approaches that support both microbial engraftment and metabolic activity have gained increasing attention. Synbiotics, combining probiotics and prebiotics, aim to enhance the survival, colonization, and metabolic activity of beneficial microbes, with effects dependent on the specific formulation [167]. Early trials in infants with CMA demonstrated that amino-acid-based formulas supplemented with *Bifidobacterium breve M-16V* and oligosaccharides shifted microbial composition toward a breastfed-like profile and alleviated allergic symptoms via IL-33/ST2 modulation [168,169].

Beyond targeted supplementation strategies, more comprehensive approaches seek to globally restore microbial community structure and function. Fecal microbiota transplantation (FMT) offers a comprehensive approach to restore eubiosis and modulate FA susceptibility in preclinical models [170,171]. Phase I trials (NCT02960074, NCT03936998) are investigating oral encapsulated FMT in peanut allergy and rationally designed microbial consortia (e.g., VE416) in combination with OIT. Overall, microbiota-targeted interventions may complement existing therapies by restoring gut homeostasis, enhancing barrier function, and modulating MC responses, though further clinical validation is required.

## 6. Conclusions

FA arises from a complex interplay between the gut microbiota, epithelial barrier integrity, and MC activation. Early-life microbial perturbations can compromise immune maturation, reduce production of tolerogenic metabolites, and weaken epithelial tight junctions, facilitating allergen translocation and promoting Th2-skewed immune responses. This establishes a self-reinforcing loop in which dysbiosis, barrier dysfunction, and MC hyperreactivity amplify one another, increasing susceptibility to allergic sensitization and severe reactions. Therapeutic strategies are evolving beyond allergen avoidance and symptom management toward interventions that target this pathogenic loop. Allergen-specific immunotherapy, biologics, and microbiota-directed approaches—including probiotics, prebiotics, synbiotics, and FMT—have the potential to modulate microbial composition, reinforce epithelial integrity, and recalibrate MC reactivity, ultimately promoting oral tolerance and reducing disease severity. In summary, the microbiota–epithelium–MC axis represents a central mechanistic framework in FA pathogenesis, providing a rationale for preventive and therapeutic strategies that restore microbial and immunological homeostasis.

## Data Availability

No new data were created or analyzed in this study. Data sharing is not applicable to this article.
